# Emergence of β-Band Oscillations in the Aged Rat Amygdala during Discrimination Learning and Decision Making Tasks

**DOI:** 10.1523/ENEURO.0245-17.2017

**Published:** 2017-10-06

**Authors:** Rachel D. Samson, Adam W. Lester, Leroy Duarte, Anu Venkatesh, Carol A. Barnes

**Affiliations:** 1 Evelyn F. McKnight Brain Institute, University of Arizona, Tucson, AZ 85724; 2 Division of Neural Systems, Memory and Aging, University of Arizona, Tucson, AZ 85724; 3 Departments of Psychology, Neurology and Neuroscience, University of Arizona, Tucson, AZ 85724

**Keywords:** basolateral complex of the amygdala, probability discounting, probability discrimination, reward expectation, reward magnitude discrimination

## Abstract

Older adults tend to use strategies that differ from those used by young adults to solve decision-making tasks. MRI experiments suggest that altered strategy use during aging can be accompanied by a change in extent of activation of a given brain region, inter-hemispheric bilateralization or added brain structures. It has been suggested that these changes reflect compensation for less effective networks to enable optimal performance. One way that communication can be influenced within and between brain networks is through oscillatory events that help structure and synchronize incoming and outgoing information. It is unknown how aging impacts local oscillatory activity within the basolateral complex of the amygdala (BLA). The present study recorded local field potentials (LFPs) and single units in old and young rats during the performance of tasks that involve discrimination learning and probabilistic decision making. We found task- and age-specific increases in power selectively within the β range (15–30 Hz). The increased β power occurred after lever presses, as old animals reached the goal location. Periods of high-power β developed over training days in the aged rats, and was greatest in early trials of a session. β Power was also greater after pressing for the large reward option. These data suggest that aging of BLA networks results in strengthened synchrony of β oscillations when older animals are learning or deciding between rewards of different size. Whether this increased synchrony reflects the neural basis of a compensatory strategy change of old animals in reward-based decision-making tasks, remains to be verified.

## Significance Statement

Aging is associated with network reorganization that can compensate for less effective functioning. Here, we examined how age impacts local circuit rhythmicity in the basolateral complex of the amygdala (BLA) during performance of discrimination learning and decision-making tasks. We found age- and task-specific increased power of β oscillations (∼20 Hz). Specifically, only aged rats showed increased power in β frequencies, which was most prominent during expectation of large rewards. Thus, aging appears to impact BLA networks in a way that promotes the emergence of β-band activity when learning or deciding between rewards of different sizes. Because β oscillations are thought to facilitate connection of distant brain regions, the enhanced β power may reflect restructuring of reward networks during aging.

## Introduction

With aging, certain cognitive domains are known to decline, notably working memory, long-term memory, and processing speed (for review, see Park and Reuter-Lorenz, 2009). A domain that is spared, however, is emotional memory (Prakash et al., 2014), which can even improve with aging. Indeed older adults remember better positive items and events that young adults do (Reed and Carstensen, 2012). Emotional memory, for negative and positive items is accompanied by changes in brain network activation, primarily consisting in increased bilateralization of the recruited brain structures (Jacques et al., 2009; Ziaei et al., 2017).

The amygdala plays a central role in emotional learning. In humans, aging impacts BOLD activity in the amygdala, but the effect depends on the elicited emotion. Indeed, viewing and rating a positive image is associated with increased amygdala activation in old compared to young adults (Mather et al., 2004), whereas viewing images of anger leads to decreased amygdala activation in older adults (Fischer et al., 2005). Because the amygdala is connected with many other brain structures (Price, 2003; Samson et al., 2005; Pessoa, 2008; Haber and Knutson, 2010), small changes in its activity has the potential to make a significant impact on brain function.

In animal models, neurons of the basolateral complex of the amygdala (BLA) are known to play an important role in detecting changes in reward value. Indeed, in young rats, BLA neurons have been shown to respond differently to rewards of different magnitudes (Pratt and Mizumori, 1998; Bermudez and Schultz, 2010; Peck et al., 2013). In aging, we have previously shown that aged rats perform differently than do young in a probability discounting task, known to depend on amygdala activity (Samson et al., 2015). While there is no report of cell loss with aging in the amygdala (Good et al., 2001), there are changes in dendritic arborization in rats that may indicate changes in synaptic connectivity (Rubinow et al., 2009), and also altered functional connectivity in humans during emotional memory tasks (Jacques et al., 2009). To elucidate how the intrinsic network activity of this structure reorganizes with aging, we recorded local field potentials (LFPs) and single-unit activity of BLA neurons while young and aged rats acquired and retrieved learned information over delays while performing reward magnitude discrimination, probability discrimination and probability discounting tasks. Because increases in gamma power coherence around (∼40 Hz), has been shown to be elicited during associative learning in cats (Bauer et al., 2007; Popescu et al., 2009) and during probabilistic learning in rats (Terada et al., 2013), we hypothesized that a similar elevation in gamma power would occur in our tasks and that differences would be found between age groups.

## Materials and Methods

### Animals and pretraining

Thirteen Fisher-344 male rats, six young (nine months) and seven old (24 months), were used in this study. These rats were acquired from the National Institute of Aging colony at Charles River. They were housed individually within a Plexiglas guinea pig container, in a facility maintained on a reversed light-dark cycle. All rats were screened regularly for health issues such as cataracts, nutrition intake, appearance of tumors, and removed from the experiment at the first signs discomfort or distress. All animal procedures were conducted in accordance with the United States National Institutes of Health Guide for the Care and Use of Laboratory Animals and approved by the University of Arizona Institutional Animal Care and Use Committee. After a period of acclimation, all rats were first tested for motor ability, vision, and spatial learning using the Morris swim task (Morris, 1984). Spatial (hidden platform) testing was administered over four consecutive days, with six training trials per day followed by visual assessment (visible platform). The visual testing was conducted for six trials per day for two consecutive days. Full details of the swim task are as described in Barnes et al. (1996). Performance on the Morris swim task was monitored using Any-maze (Stoelting) and calculated using a corrected integrated path length (Gallagher et al., 1993) for all spatial and visual trials. A CIPL measure was used to control for the variability in individual differences in swimming speed and release locations.

After the Morris swim task, rats were food deprived to 85% body weight and trained to press two levers for food reinforcement (vanilla-flavored Ensure). The shaping and training procedure is described in Samson et al. (2014, 2015). Briefly, once rats were able to press levers, they were trained for seven consecutive days, at a predefined probability schedule to reinforce their lever press performance. The reward schedule employed approximated a random ratio (RR) reinforcement schedule, to increase the number of lever presses needed to obtain a reward. Specifically, days 1–3 were performed at RR1, days 4–5 at RR2, and days 6–7 at RR3. Each training session lasted until the rat reached 50 rewards or 40 min. There were two daily training sessions, one for each lever.

### Apparatus, operant chamber

Training and testing were conducted in trapezoid shaped stainless steel (62 × 32 × 39 cm) chambers (Fig. 4*G*), as described in Samson et al. (2015). These chambers were equipped with two retractable levers (Med-Associates), a cue light above each lever, an infrared beam (Med-Associates) to record head entry into the food area, a food reward port controlled by a solenoid valve (Parker Hannifin), a sound attenuating shell around the operant chamber and speakers delivering white noise. Control of the experimental procedures was conducted by a computer interfaced with the behavioral testing chambers using Basic-X software (NetMedia). Task and behavioral events were timestamped by parallel outputs from the Basic-X into the Cheetah acquisition system (Neuralynx).

### Surgical and electrophysiological recording procedures

Surgery was conducted according to National Institutes of Health guidelines for rodents and approved Institutional Animal Care and Use Committee protocols. The rats were implanted, under isoflurane anesthesia, with a “hyperdrive” array of 12 separately moveable tetrodes. Tetrodes were constructed of four polyimide-coated nichrome wires (13 μm in diameter) that were twisted together. For all rats, amygdala recordings were performed in the left hemisphere (3.0 mm posterior to bregma, 5.0 mm lateral to the midline). The 12 tetrodes were placed at a depth of ∼1.0 mm at surgery and were slowly moved ventrally over the next 14 d to record spikes extracellularly from the BLA (starting at ∼7.0 mm from the brain surface). Two additional tetrodes, with wires shorted together, served as references and were lowered to a cellular “quiet area” of the brain, ∼3.0 mm from the brain surface. In addition, two skull screws were placed above the cerebellum, for post-recording LFP rereferencing purposes. After recording began, tetrodes were lowered at the end of most recording sessions by 39.5 µm. Tetrodes were lowered between consecutive days on the same task (reward size discrimination, probability discounting or probability discrimination).

On the last day of electrophysiological recording, a small current pulse (15 µA, 10 s) was applied to each tetrode channel to create a small iron deposit thereby marking the location of the tetrodes end points. On the following day, rats were perfused with 4% PFA, brains were extracted and the tetrode end points revealed using standard histologic procedures with Nissl and Prussian blue staining (Fig. 2*B*).

Each tetrode was attached to four separate channels of a 54-channel unity–gain head stage and was connected via shielded wires to programmable amplifiers (Neuralynx). Video data were obtained via LEDs mounted on the headstage and tracked using a black-and-white CCD camera mounted to the sound attenuating chamber. Video tracking was recorded with a sampling frequency of 60 Hz, and a spatial resolution of 0.2 cm/pixel. Spike signals were amplified (500–5000 V/V), bandpass filtered (600 Hz to 6 kHz.), and digitized (32 kHz) using a Cheetah Data Acquisition System (Neuralynx). Events that reached a custom-set threshold (typically 5–20 μV) above the level of baseline noise were recorded for a 1-ms duration. EEG signals were bandpass filtered between 1 and 300 Hz, sampled at 1.871 kHz, and amplified on the head stage with unity gain, and then again with variable gain amplifiers (up to 5000).

### Behavioral procedures

**Figure 1.**
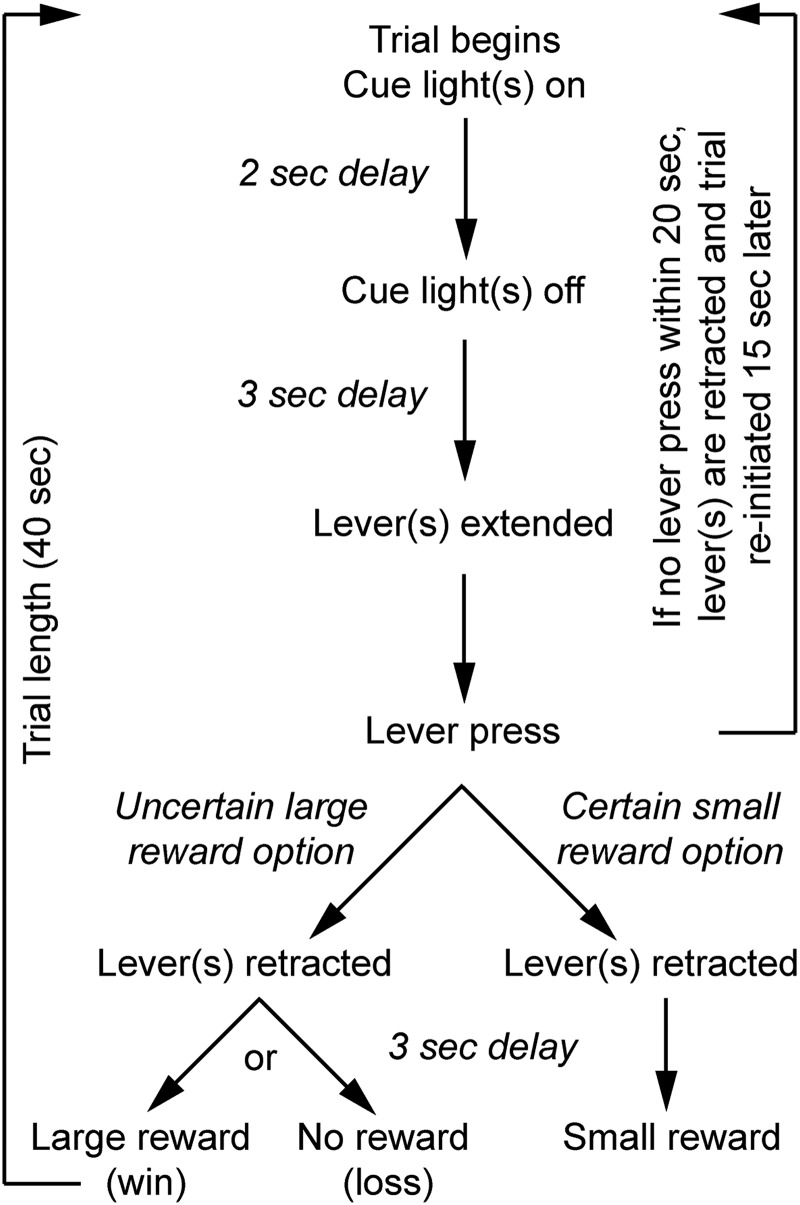
Trial design. Timeline of trials on the probabilistic discounting task. A trial began with a 2-s cue-light on, above the respective lever. Two seconds after cue light was turned off, one or two levers were extended for the rat to press. Failure to press within 20 s resulted in lever retraction and the trial was reinitiated 15 s later. Pressing the lever for either large/uncertain or small/certain rewards led to lever retractions and reward delivery 3 s after lever press (loss trials resulted in an absence of reward delivery). For the reward magnitude discrimination task, the choices were small and variable sized rewards. For the probability discrimination task, the choices were certain and uncertain rewards (as depicted in Fig. 2). The next trial was initiated 40 s after the past cue-light-on event.

All daily sessions consisted of three phases, a pre-task rest phase of at least 30 min, an experimental task phase and a post-task rest phase of at least 30 min. Four different tasks were performed after surgery; a lever discrimination training, a reward magnitude discrimination task, a probability discounting task and a probability discrimination task. The overall task structure was the same and is as follows: a predetermined number of trials were separated into five blocks, each starting with forced choice trials followed by free choice trials. Forced choice trials are defined as trials in which only one lever is available for the rat, whereas on free choice trials both levers are available for the rat to choose. The order of lever presentation during forced choice trials was pseudorandomized and lever-reward associations were counterbalanced between animals. The general trial structure is shown in Figure 1. Trials began with cue light(s) on for 2 s followed by a 3-s delay after which lever(s) were extended into the chamber. Rats had 20 s to press and failure to press within that time resulted in the retraction of the lever(s) and reinitiation of the trial 15 s later. A lever press resulted in a lever retraction(s) and outcome delivery 3 s later (reward: “win trials” or nothing: “loss trials”). The next trial began after 40 s had passed since the cue light(s) signaled the start of a trial. The number of trials per block differed between each task. For the lever discrimination training and the reward magnitude discrimination task, each block consisted of eight forced choice trials (four per lever) followed by six free choice trials. For the probability discounting and probability discrimination tasks, each block consisted of 16 forced choice trials (eight per lever) followed by 10 free choice trials.

**Figure 2.**
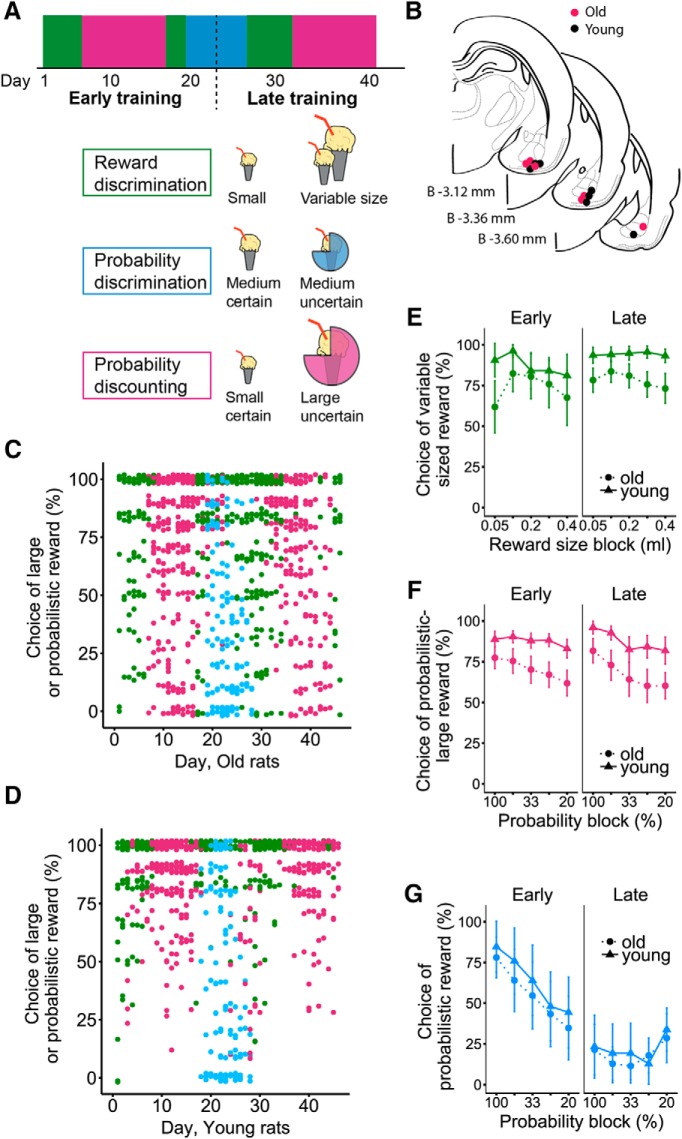
Behavioral performance of young and aged rats during discrimination learning and decision-making tasks. ***A***, Schematic illustrating the pattern of alternation between the three decision-making tasks. For analysis purposes, the training periods are separated into early and late periods. Cartoon representing each task: the reward discrimination task (green) involved choosing between a small and a variable sized larger reward, the probability discounting task (magenta) involved choosing between a small/certain reward and a large/uncertain reward and the probability discrimination task (blue) involved choosing between medium/certain reward and a medium/uncertain reward. ***B***, Schematic of coronal sections based on Paxinos and Watson (2014), showing the marker lesion sites at the endpoints of the tetrode recording probes on the last day of the experiment, for each rat. Tetrodes were lowered at the end of each recording day for the young (black filled circles) and old (red filled circles) groups. Analyses only include days in which tetrodes were located within the BLA. ***C***, Percentage choice of the variable sized rewards and uncertain rewards across days of training in old rats. Scatterplots of the behavioral performance of each rat across days of training (*x*-axis), in the three tasks (as described in ***A***). Performance values are jittered vertically to prevent overlap. ***D***, Same as ***C***, in young rats. ***E*,** Percentage choice of the variable sized reward over blocks of trials (*x*-axis), during the early (solid line) and late (dashed) training phases, for the reward magnitude discriminations task. Symbols represent mean ± 95% CI. There is a significant effect of age in the late training phase (*p* < 0.05). ***F***, Percentage choice of the large/uncertain reward over blocks of trials (*x*-axis), during the early (solid line) and late (dashed) training phases, for the probability discounting task. Symbols represent mean ± 95% CI. There is a nonsignificant trend for an age difference (*p* = 0.08). ***G***, Percentage choice of the uncertain reward over blocks of trials (*x*-axis), during the early (solid line) and late (dashed) training phases, for the probability discrimination task. Symbols represent mean ± 95% CI. There is no significant age difference (*p* > 0.05).

Two weeks after surgery, rats were habituated to the training apparatus and to the novel task design using either a lever discrimination training (four young and four old rats) or the reward magnitude discrimination task (two young and three old rats). In the lever discrimination training, the size of rewards was equal (0.05-ml vanilla Ensure) for both levers and 100% certain. For the reward magnitude discrimination task, one of the levers was associated with a variable sized reward, monotonically increasing from 0.05–0.4 ml of vanilla Ensure (0.05, 0.1, 0.2, 0.3, 0.4 ml; Table 1; Fig. 2*A*). The block order was generally reversed every 3 d, between small to big reward and big to small reward. For analyses, the data from the two block order versions are collapsed, as statistical analyses did not reveal differences. The reward magnitude discrimination task was the first task performed with neural recordings (early training phase; Fig. 2*A*), and was administered a second time in the experiment (late training phase; Fig. 2*A*), so that the neural data on this task could be sampled at different dorsoventral axis of the BLA. A third set of training on the reward magnitude discrimination task was also performed at the end of the experiment, but these data are not included in the analyses because our histologic assessment indicates that the electrode tips had passed beyond the BLA in some rats.

**Table 1.** Experimental parameters for the reward discrimination, probability discounting, and probability discrimination tasks

Task	Reward options	Probabilities (%)	Reward (ml)
Reward magnitude discrimination	Variable size	00	0.05–0.4
	Fixed small	100	0.05
Probability discounting	Uncertain/large	0–100	0.2
	Certain/small	100	0.05
Probability discrimination	Uncertain/medium	0–100	0.1
	Certain/medium	100	0.1

For the probability discounting task, the reward size was 0.05 and 0.2 ml, respectively, and the larger reward option was probabilistic (Table 1; Fig. 2*A*). This experiment was performed for 20 d, separated into two set of 10 consecutive days (early and late training phases; Fig. 2*A*). There were two versions of this task, one in which reward probabilities monotonically decreased from 100% to 20%, and in the other, reward probabilities monotonically increased from 20% to 100%. For analyses, the data from the two versions are collapsed, as statistical analyses did not reveal differences. Only 9 d in the late training phase are included in the analyses because the electrode tips on day 10, on some of the rats, were considered ventral to the BLA (the dorsoventral tetrode end location is a conservative estimate).

For the probability discrimination task, the size of reward was made equal for both levers (0.1 ml) and the probability of receiving reward was varied (Table 1; Fig. 2*A*). This task was performed halfway through the experiment (Fig. 2*A*) and consisted of six consecutive days; 3 d in which the reward probabilities were monotonically decreased from 100% to 20%, followed by 3 d in which the reward probabilities monotonically increased from 20% to 100%. It is important to note that the reward probabilities were set using a random number generator, such that the probability of receiving a reward on a given trial is independent of the outcome from the previous trials. Analyses were performed two ways, by using the administered probability and by using the actual experienced probability, for each block of trials.

### Data analysis

#### Analysis of behavioral data

Analyses of the behavioral data were performed in R (R Development Core Team, 2013). Behavioral performance was analyzed using a rank-based statistical model for repeated measures, with the nparLD package in R (Noguchi et al., 2012). Nonparametric statistics were used here because implanted young rats preferentially pressed for the large reward, thereby creating a strong deviance from normality. This method computes an ANOVA-type statistic (ATS) to assess the main effects of age and trial blocks, and their interaction.

#### Analysis of neural data

##### Spike sorting

Spikes were sorted offline by first using the automated spike-sorting algorithm KlustaKwik (K. D. Harris, University College, London, United Kingdom) to isolate units and separate them from noise. The resulting clusters were then refined manually using cluster cutting software (MClust 3.2; A. D. Redish, University of Minnesota, Minneapolis, MN, with customizations by P. Lipa, University of Arizona, Tucson, S.L. Cowen, University of Arizona, Tucson, AZ and D. Euston, University of Lethbridge, Canada), resulting in spike-train time series for each of the well-isolated cells. Only units with <0.3% interspike intervals (ISIs) falling within 2-ms refractory period and with a minimum of 0.01-Hz firing rate during the pre-task rest period were included.

##### Analysis of single-unit data

Neuron classification was based on the firing dynamics using a 500-ms single-sided autocorrelation (AC, with 1-ms bin size) to calculate the decay rate, a smoothed log transformed ISI distribution to detect bimodality and the local variance (LV). Specifically, tonic firing neurons were identified as neurons with LV < 0.5, irregular firing neurons as neurons with an AC decay rate lower than −0.005, irregular/bursty neurons had a decay rate higher than −0.005, and bursty neurons were all neurons with bimodal log ISI distributions.

##### Analysis of local-field potentials

Only LFP data from a single tetrode for a given session were included. Analyses were performed on both the original LFP data and the rereferenced LFP data to a cerebellar screw. To reduce the impact of high-frequency artifacts on the local field signal from action potentials (Ray and Maunsell, 2011), local field activity was first “de-spiked” by removing a 2-ms window of data around spikes and replacing the absent data using cubic spline interpolation (Zanos et al., 2011). The de-spiked LFP traces were then filtered with a 15–30 Hz fourth-order Butterworth bandpass filter (second order applied in forward and backward directions for zero phase distortion). To examine β and theta frequencies, spectral LFP data were computed using the discrete Fourier transformation on each trial, using the time window following each lever press (0.1–2.9 s post-lever release). Power difference was calculated by subtracting baseline power (−2.9 to −0.1 s before light on, indicating trial onset), from power post-lever release. For simplicity, we refer to lever press in the text, specifically, the time windows for analysis is based on lever release (i.e., removal of the paw from the lever). β Power differences across tasks, trials or blocks was assessed using Kruskal-Wallis rank sum statistics, Pearson product moment correlations and Bonferonni corrected.

To assess whether β power was different across location within the BLA or outside of the BLA, differences in β power across all tetrodes of five aged rats was examined. For this analysis, we used four sessions of probability discounting task and the first 30 trials, thus restricting for trials with the highest β power. Power in each tetrode was normalized by subtracting the mean power across tetrodes, within a recording session. Differences in β power across tetrodes was also assessed using the Kruskal-Wallis rank sum statistics.

##### Identification of β events

To extract β events, de-spiked signals were bandpass filtered (20–40 Hz) and squared. A candidate β event was identified when the squared power exceeded 2 SDs above the mean and lasted for at least 350 ms. False positives caused by clipping or other signal artifacts were manually rejected. β Events were used to compute the incidence of β events within trials (number of β events/number of trials).

##### β incidence analysis

Initial analyses of the change in β incidence was calculated using a linear mixed-effects models (LMMEs) to assess the effect of age, trials, days and any interaction (Samson et al., 2015, 2014). We used the lme4 and nlme packages to perform our mixed-effect model analysis in R (Bates et al., 2015; Pinheiro et al., 2017; R Development Core Team, 2013). Our starting model was as follows: Yit = α + β1Ageit + β2Dayit + β3 (Ageit * Dayit) + ai + biDayit + εit

In this model, Y_it_ was the dependent variable, either average percentage of lever presses for the large reward associated lever, for animal *i* on day *t*. α represents the average-dependent variable start value (baseline performance level). β_1_Age_it_ and β_2_Day_it_ represent the average age and day effects on the slope of the model. β_3_ (Age_it_ * Day_it_) represents the interaction of age and day. a_i_ and b_i_Day_it_ represent the random variation in the intercept and slope of the model, and ε_it_ represents the residuals. All random variables were assumed to have a zero mean and a Gaussian distribution. Model selection, to assess the significance of each variable, was performed using the maximum likelihood method. The χ^2^ values, degrees of freedom and *p* values resulting from the likelihood ratio test are reported in the results section. Normality in the distribution of the residuals of final models was assessed using scatter and quantile-quantile plots.

##### Spike-LFP coherence analysis

To extract the phase of β during which single neurons fired maximally, spikes were first restricted to β epochs (as defined above). Then, 50 randomly selected spikes were considered. Cells with <50 spikes within β epochs were discarded from this analysis. These contingencies preserved consistent sampling across cells and sessions. Spike-β LFP phases were derived from the Hilbert transformed β LFP signal and were represented as normalized vectors. Statistics for the firing rate modulation by β oscillations was performed using the robustfit function of Matlab, which creates a robust multilinear regression. Coherence statistics were performed using Matlab and the Rayleigh *z* test. $z=nr^{2},$where r is the magnitude of the mean vector and n is the number of spikes during β epochs. Significance was established using a *p* value estimated by exponential fit, accounting for the Rayleigh distribution of the data

$$p\approx e^{\sqrt{1+4n+4\left(n^{2}-(n{R)}^{2}\right)}-(1+2n)}$$

##### Localization of β events in the operant chamber

The location of high-β power events within the operant chamber was determined using the pixel values of the video recording. Because of light interference entering the operant chamber, a cubic spline interpolation of the video data, followed by a hamming window convolution (500 ms), was necessary to infer location of the rat within the operant chamber during periods of high light noise. Sessions with >40% of invalid spatial timestamps were discarded from spatial analyses (6.63%, 35/528 sessions).

Reward zone occupancy was calculated by adding all position samples within a 10 × 20 cm area in front of the reward zone during trials with valid lever presses. To normalize the position data for occupancy, the video frame was subdivided into a 24 × 30 matrix (where each grid is 20 × 20 px) and each bin was assigned a measure of time spent in a bin. The normalized position was used to create a spatial bivariate histogram of subsampled (60 samples/s to match position video samples) β LFP power. The β filtered LFP data were subsampled to match the position sample rate. For aesthetic reasons, the histogram is smoothed and represented as a heatmap (Fig. 4*D*).

Rat movement velocity was inferred from the Euclidian distance traveled over 0.5 s following the onset of a β event. As a control, movement during task-evoked β events was compared to the movement during a baseline 0.5-s interval taken before the light cue indicating trial onset. This baseline period was used as a “low-movement” comparison condition for the “high-movement” period during which β oscillations typically occurred in this task, corresponding to rats reaching the goal location.

## Results

### Behavioral performance

Ensemble neural activity and LFPs were continually recorded, while young (*n* = 6) and aged (*n* = 7) rats acquired and performed a series of decision-making tasks. The rats had never experienced the decision-making task until the surgical implantation of the hyperdrive apparatus with independently controlled tetrode recording probes. Animals only received operant lever press training before the surgery. To acclimate to the weight of the hyperdrive apparatus, rats either performed a lever discrimination training (four young and four old rats), or reward magnitude discrimination task (two young and three old rats) until they were able to complete a task session (which required 3–5 d). The behavioral analysis presented here is from the onset of the neural recordings and includes all days recorded within the BLA.

As shown in Figure 2*A*, the different decision-making tasks were performed in one or two series within the experiment. This was to allow sampling of the neural activity on each task at different dorsoventral regions of the BLA (see Materials and Methods). Because this electrophysiological experiment targeted the change in neural activity during the acquisition phase of a decision task, as rats learned the value of options, the behavioral results complement the ones we previously published using these tasks (Samson et al., 2015). For analysis purposes, behavioral performance was assessed separately for the early and late training phases (Fig. 2).

Overall, aged rats tended to choose more often the small reward option over the large or the probabilistic one (Fig. 2*C*,*E*) and statistics were performed for each task individually. The reward magnitude discrimination task was performed over two series of 3–5 d (Fig. 2*E*). All rats began the recording portion of the experiment by performing five consecutive days of testing on the reward magnitude discrimination task, referred to as the early training period. In this period, we found no effect of age or reward magnitude on performance (age: *F*_(1,∞)_ = 1.37, *p* > 0.05; reward: *F*_(1.6,∞)_ = 1.43, *p* > 0.05) suggesting similar levels of discrimination performance, early in training. A second testing period on the reward magnitude discrimination task was performed halfway through the experiment, and we refer to it here as “late training.” We found an age effect on performance (*F*_(1,∞)_ = 12.30, *p* < 0.01), with no effect of reward magnitude (*p* > 0.05), for the late period reward magnitude discrimination task. This suggests that aged rats select more often the small reward across magnitude blocks than do young rats.

For the probability discounting task, two series of 10 consecutive session days were performed, one in the early and the other in the late phase of training (Fig. 2*F*). In the early phase of training, there was a significant effect of probability block (*F*_(2.99,∞)_ = 6.86, *p* < 0.001), but not of age (*F*_(1,∞)_ = 2.49, *p* > 0.05). For the late phase of training, there was a tendency toward an interaction effect (*F*_(2.23,∞)_ = 2.34, *p* = 0.08), an effect of probability blocks (*F*_(2.32,∞)_ = 16.30, *p* < 0.001), but not of age (*F*_(1,∞)_ = 0.32, *p* > 0.05) on performance. These results suggest that with training, aged rats developed a tendency to press the lever more often for the small/certain reward.

The probability discrimination task was performed over six consecutive days, halfway through training. The analyses were divided into the first 3 d and the second 3 d on this task. For both groups of days (Fig. 2*G*), there was a significant effect of probability block (early: *F*_(2.37,∞)_ = 17.42, *p* < 0.001; late: *F*_(1.97,∞)_ = 4.89, *p* < 0.05) on the performance of both age groups, with no age effect (early: *F*_(1,∞)_ = 0.01, *p* > 0.05; late: *F*_(1,∞)_ = 0.002, *p* > 0.05). These data suggest that both young and aged rats learned to avoid the probabilistic reward, compared to the certain reward, similarly over the 6 d of training.

To further describe the source of the increased selection of the small/certain reward in the late training period of the probability discounting task, we performed a Win/Stay-Lose/Shift analysis. Consistent with earlier work (Samson et al., 2015), aged rats showed increased lose-shift responding as compared to young rats (Kruskal-Wallis, χ^2^(1) = 12.9, *p* < 0.005). This is suggestive of an increased sensitivity to negative feedback (i.e., losses), in aged rats, leading to an age-related increase in risk averse behavior on this decision-making task.

### Impact of aging on oscillatory activity in the amygdala

**Figure 3.**
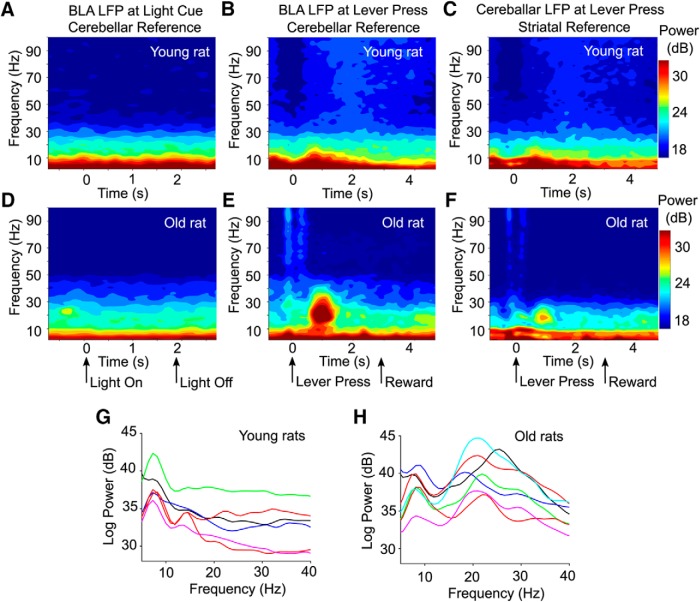
Significant β-band oscillation (15–30 Hz) power increase in old rats after lever presses. ***A***, Normalized spectrogram of an example recording session from a young rat, with 0 being aligned to light cue onset. For this example, the recording electrode was located in BLA and the signal was rereferenced to a cerebellar screw in the skull. The light cue was 2 s in duration (indicated by the arrows below ***D***). ***B***, Normalized spectrogram of the same rat and recording session as in ***A***, with 0 being aligned to the lever press and the signal rereferenced to a cerebellar screw. Reward delivery occurred 3 s after lever press (indicated by the arrows below ***E***). ***C***, Same spectrogram time point as for ***B***. Signal is taken from the cerebellar LFP recording screw which reflects the signal between it and the reference tetrode located in a quiet area in the fiber bundle dorsal to posterior striatum. ***D***, Same as ***A***, for an old rat. ***E***, Same as ***B***, for an old rat, taken from the same rat and session as in ***D***. Note the elevated power in the β range, around 15–30 Hz. ***F***, Same spectrogram time point as ***E***. Signal is taken from the cerebellar LFP recording screw which reflects the signal between it and the reference tetrode located in a quiet area in the fiber bundle dorsal to posterior striatum. Note the attenuated signal in the β-range frequencies (15–30 Hz) near posterior striatum as compared to that of the BLA LFP signal in ***D***. ***G***, Power spectra from all young rats during the first session of the late training phase of the reward discrimination task. Average session spectra taken during the period after a lever press (0.3–2.0 s post-lever press), showing an elevated theta peak, but not β in the young rats (each color represents the power spectra of a different rat). This is with the exception of one young rat showing elevated power around 15 Hz. ***H***, Same as in ***E***, for all aged rats. Figure shows elevated power in the theta and β ranges in all aged rats.

We first performed a spectral analysis of the LFP activity to assess whether any age differences exist during task performance. We found a pronounced increase power in the β-band range (15–30 Hz) in aged rats (Fig. 3*D*,*E*,*H*), which was not present in young rats (Fig. 3*A*,*B*,*G*). This period of high power at β-band frequency occurred at the time point between the lever press actions and reward delivery, as shown by the session averaged spectrogram (Fig. 3*D*). To assess this effect statistically, the raw β power in all rats was measured before and after lever presses and a significant interaction effect between age group and β power was found (*F*_(1,∞)_ = 21.06, *p* < 0.0001). To confirm that β oscillations did not occur as a result of the rereferencing of the LFP to cerebellar screws, we compared β power between the original (BLA signal referenced to a tetrode located in the fiber track dorsal to posterior striatum) and rereferenced recordings and found similar β power. In addition, we inferred β power at the reference tetrode using the cerebellar screw recording and found β power at the reference tetrode to be about a third of the power recorded at BLA tetrodes (33 ± 14%; Fig, 3*C*,*F*, for example of the reference spectrogram). These results suggest that the β oscillations recorded in BLA in this study, do not originate from an area close to our reference electrode such as the posterior striatum.

**Figure 4.**
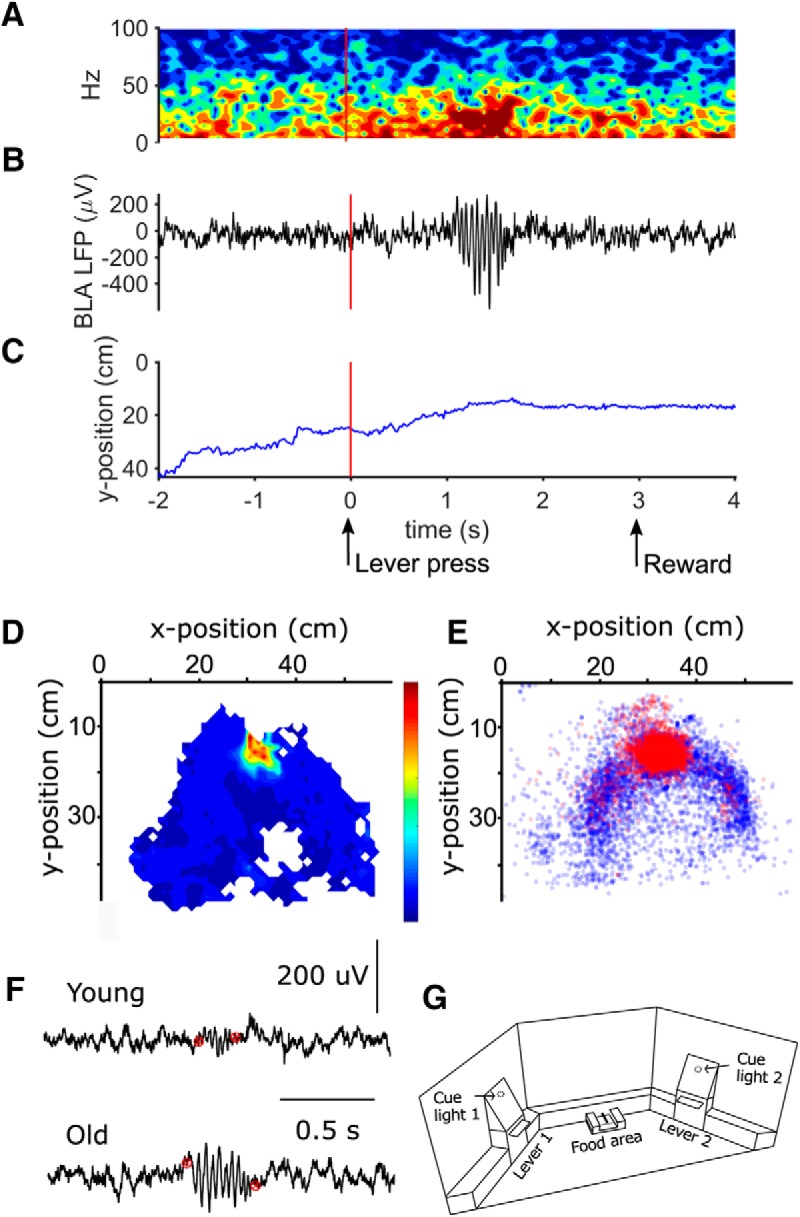
β Epochs occur primarily when old rats arrive at the reward zone. ***A***, Example of a single trial spectrogram after a lever press. The vertical red line at 0 indicates lever press. Hot colors indicate elevated power. Vertical red lines denote the lever press (***A****–****C***). ***B***, LFP trace corresponding to the spectrogram in ***A***, showing a burst of β activity. ***C***, *y*-position of the rat at the time of the LFP recordings in ***A***, ***B***. The blue line represents the position along the *y*-axis, as the rat goes toward the feeder (see *y*-axis depiction in ***E***). The arrow at 0 s indicates lever press, and the one at 3 s indicates reward onset. ***D***, Example of a session from an old rat. Shown is the average normalized heat map of β power weighted by the percentage time spent in that location. The color bar represents the gradient change in normalized power, warmer colors indicate the location with highest β power, and a lack of color indicates positions that were not covered by the rat during the session. The heat map in this panel is oriented similarly to the operant chamber illustration in ***G***. ***E***, Spatial location of β events of all aged rats (all sessions). The location was determined based on the position of the rat’s head at the start of each β oscillation, as shown in ***F***. Red circles indicate β oscillations occurring between lever press and reward time points, whereas blue circles indicate all other β oscillations that occurred during the experimental tasks. ***F***, LFP traces showing representative example bursts of β activity in young and old rats. The red markers indicate the start and end time points of each β burst. These time points were used to identify trials containing task-evoked β oscillations. Note that when present, β oscillations in young rats were of lower amplitude than those from the aged rats. ***G***, Schematic drawing of the operant chamber used for the discrimination learning and decision-making task. The chamber is of trapezoid shape with small walls on the side of the chamber to minimize potential collisions between the brain implant and the chamber walls.

To further characterize β oscillations in all animals, we analyzed the power spectrum between 5 and 40 Hz more carefully, using the first session of the reward magnitude discrimination task of the late-training period. In this session, we found that in aged rats, the peak frequency in the β range varied from 19.2–25.6 Hz (Fig. 3*G*). There was no clear β peak in the younger animals (Fig. 3*H*). When present, β oscillations in young rats were of low amplitude (Fig. 4*F*). For comparison purposes, we also examined whether theta oscillations (6–10 Hz) were different between age groups in the BLA, after lever presses, when β oscillations are maximal. For the reward magnitude discrimination task (late period), theta was faster in aged rats (old: 7.7 ± 0.2 Hz; young: 7.2 ± 0.1; Kruskal-Wallis, χ^2^(1) = 4.011, *p* = 0.0452) and theta power was also different between age groups (interaction effect, *F*_(1,∞)_ = 5.35, *p* < 0.05). Specifically, theta power was greater during the baseline period before lever press (−2.0 to −0.3 s prelever press) in young rats and greater after lever press in aged rats (0.3–2.0 s post-lever release). Because spectral analyses show the strongest age difference in β frequencies (Fig. 3), the focus of this manuscript will be on these oscillations.

To get better insight into the origin of β oscillations, we further examined how β power differed across tetrodes located in the BLA in contrast to neighboring structures. To this end, we compared the differences in β power across all recording tetrodes of 5 aged rats, averaged over 5 sessions of probability discounting tasks. Tetrode locations span medio-laterally from the medial nucleus of the amygdala, to 1 tetrode located immediately lateral to the external capsule (thus outside of BLA). Within rat, the medio-lateral span was around 1.5 mm for all rats and the dorsoventral span ranged from 0.6mm to 1.5 mm. We found a nonsignificant trend for β power to differ between tetrodes (Kruskal-Wallis, χ^2^(11) = 19.12, *p* = 0.058). There was, however, no relationship between β power difference and medio-lateral position (Spearman’s rank, *r*_S_ = −0.23, *p* = 0.08), nor between β power and the dorso-ventral position within the BLA (Spearman’s rank, *r*_S_ = 0.11, *p* = 0.39). Thus, the increased β power observed after lever press is not confined to BLA, as it extends to more medial and ventral regions of the amygdala. β Power recorded at the tetrode located outside of the BLA (lateral to the external capsule, or ventral to the BLA) did not differ from the other tetrodes (Within rat Kruskal-Wallis, χ^2^(11) = 11, *p* = 0.44). β Power recorded at the tetrode located lateral to the external capsule may have resulted from volume conduction as it was in very close vicinity to the BLA. The brain regions ventral of the amygdala includes the postcortical amygdala and the rostral amygdalopyriform area. Our electrodes were never positioned lateral enough to end in the piriform cortex, which is an area known for β oscillations during olfactory discrimination learning (Cohen et al., 2015).

### Location of β events within the operant chamber

As described above, the timing of task-evoked β events suggests that these primarily occur as rats reach the reward zone (Fig. 4). Because β events have previously been associated with olfactory learning (Kay et al., 2009), we further assessed whether something salient, such as the odor of the food cup, triggered β events instead of a top-down process such as expectation. To this end, we investigated the spatial distribution of spontaneously evoked β epochs. We found that spontaneous events of high β power occur in a V-shaped pattern in the operant chamber and are not confined to the reward zone (Fig. 4*E*). In addition, we tested the possibility that the higher incidence of β epochs in aged rats over young rats was due to spending more time near the reward zone. Contrary to this hypothesis, however, we found that young rats spend significantly more time at the reward zone than do aged rats, 62.4 ± 21% vs 43.41 ± 15%, respectively (Kruskal-Wallis χ^2^(1) = 80.66, *p* < 0.02e-14). These results also support the notion that β events do occur preferentially at the reward zone and by the walls of the operant chamber.

### Temporal occurrence of β events

**Figure 5.**
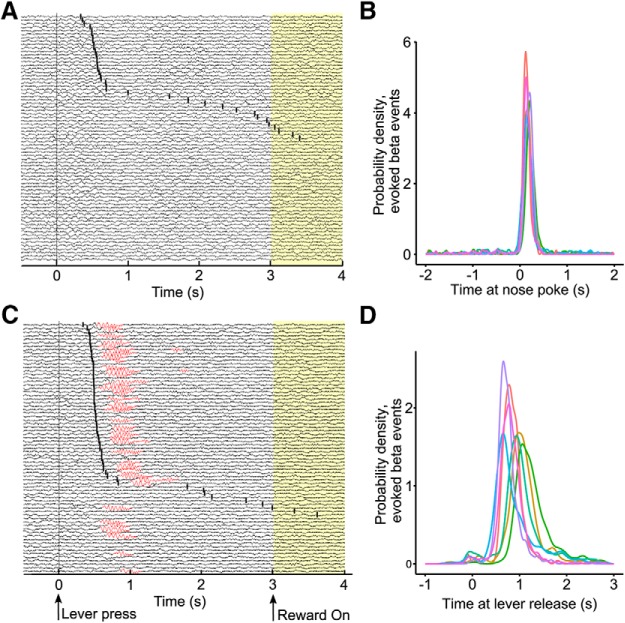
β Oscillations do not correlate with reaction times, but with nose pokes in the food area. ***A***, Young rat example of LFP traces recorded over the 70 session trials, from a reward magnitude discrimination task, sorted by reaction time (nose poke at the reward zone, black tick mark). Yellow background indicates reward delivery at 3-s post-lever release (delivery duration: 0.5 s for small, 0.5–2 s for the variable magnitude reward). ***B***, Probability density function of the timing of β epochs aligned at 0 at the nose poke in the food area, for each aged rat (denoted by the different colors). ***C***, Same as ***A***, in an old rat. Red portion of traces indicate β-band oscillation occurrence. ***D***, Same as in ***B***, with the data aligned at 0 at the lever press.

Our spectral analyses suggest that β events occur primarily between lever presses and reward delivery (Figs. 3, 4). Indeed, we found that 75% (5157/6899 β events) of β events during the task occurred within 3 s after a lever press in old as compared to 29% (1104/3786 β events) in young rats. Because both reaction times (Leventhal et al., 2012; Pollok et al., 2014) and goal-reaching (Howe et al., 2011; Lemaire et al., 2012) have been associated with increased β power, we further examined the timing relationship between β occurrence and task events, notably lever release and nose poke in the food area (Fig. 5). We found that the timing of task-evoked β epochs is almost completely time-locked to the nose poke, as shown by the narrower probability density function following nose poking as compared to lever release (Fig. 5, compare *B*, *D*). This relationship was not completely systematic, as 13% of β events occurred over 0.1 s before or over 0.5 s after nose poke (699/5157 task-evoked β events). The timing of β events with respect to lever release, however, differed across rats and the difference can be accounted for by the difference in reaction times from releasing the lever to reaching the nose poke (Pearson's product-moment correlation on rat mean reaction times: *r*(5) = 0.81, *t* = 3.1, *p* < 0.05). These results thus support the hypothesis that reaching the goal is an important variable linked to β occurrences, at least under our experimental protocol.

### Change in β oscillations over days and within session

**Figure 6.**
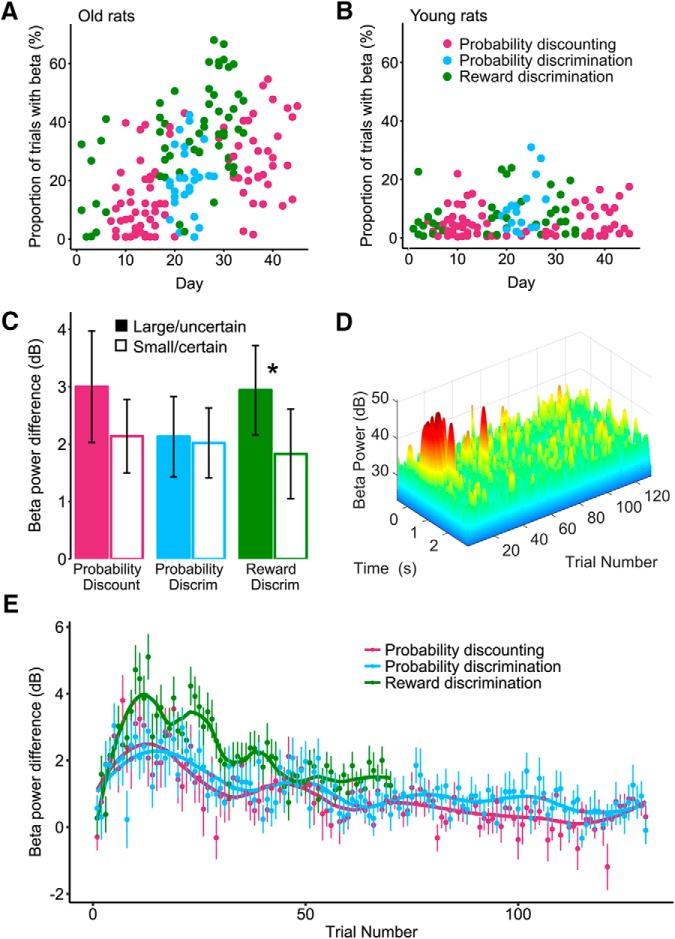
Incidence of high-power β epochs increases with training days but decreases within session trials. ***A***, The proportion of trials with task-evoked β oscillations increases across daily sessions in old rats. Colors represent the task performed: magenta, probability discounting; green, reward discrimination; blue, probability discrimination. ***B***, Same scatterplot as in ***A***, showing that β power did not increase over days in the young rats, and was, overall, lower than for the older animals. ***C***, β power is greater following presses for the large reward in the reward magnitude discrimination task in aged rats (data taken from block 1 in the late training period, where β is highest). Star denotes significance (*p* < 0.05). ***D***, Example distribution of β power over trials in a daily session of a probability discounting task illustrating the greater amount of β power events early in the task, in an old rat. The time axis is aligned at 0 for the lever press. Warmer colors correspond to higher β power. ***E***, β power as a function of trial, separated by task type (data taken from the late training period, where β is highest). Overall, β power was higher in the reward magnitude discrimination task (green). Within a session, β power is highest around trial 10 and then gradually decreases thereafter.

In associative learning tasks, Lemaire et al. (2012) have shown that dopamine-depleted rats develop β oscillations over the course of training. As shown in Figure 6*A*,*B*,*C*, we found that the incidence of task-evoked β events increased with session day (LMME; interaction effect: day by age; χ^2^(1) = 6.89, *p* < 0.01) and was influenced by the type of experiment performed (LMME; interaction effect: experiment by age; χ^2^(2) = 49.0, *p* < 0.01). These data suggest that the incidence of β oscillations is different for young and old rats across days and across types of behavioral tasks. Because of the large difference in β incidence between old (mean incidence 22.1 ± 2.3) and young rats (mean incidence 4.8 ± 1.9), the remainder of the statistical analyses will focus on the aged rats only to assess the behavioral correlates of β in the BLA.

**Figure 7.**
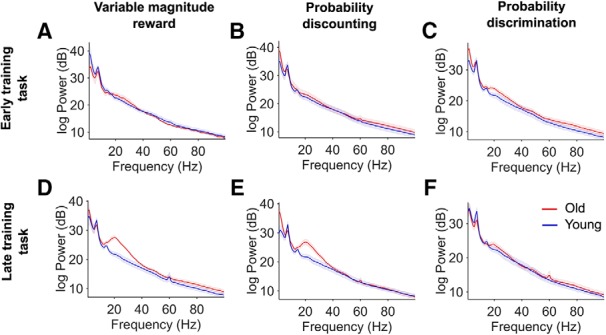
Task average power spectra in all young and aged rats, separated by training period. Each task is separated into a column, with the reward magnitude task (left), the probability discounting task (center), and the probability discrimination task (right). ***A–C***, Spectrogram of the average LFP power during task (0.1–3.0 s after lever press), for the early training period. ***D****–****F***, Same as ***A–C***, for the late training period. The blue line corresponds to the power spectra in young rats and the red line corresponds to aged rats. Shading corresponds to SEM. Note the increased power in the β-band range in aged rats, for the late training period of reward magnitude and probability discounting tasks.

We then further characterized the impact of experiment type on β incidence over days, and found that within the aged rat group, there was an effect of day and experiment type on the incidence of task-evoked β epochs (LMME; day; χ^2^(1) = 8.3, *p* < 0.01, experiment; χ^2^(1) = 53.32, *p* < 0.01). The incidence of β is greatest in the reward magnitude task, next largest in the probability discounting task and lowest in the probability discrimination task. Increase in β power in the late training periods is also shown by the separate spectral analyses in Figure 7. In the average reward discrimination task (late-training period), the average β frequency peak in old rats is 20.1 Hz. Because there was no difference between β power across tetrodes (see results above), changes in β incidence over days are likely independent of the dorsoventral location of the recording tetrodes, within the BLA.

While the overall incidence of β increased over days, the incidence of task-evoked β and power in this frequency band was greatest in the early trials of each daily session (Fig. 6*D*,*E*). To characterize this effect, we assessed the change in β power over all recording sessions collected in the late training period in each task, which are days selected for their higher incidence of β events. Overall, β power was greater for the reward magnitude discrimination task than for the two other tasks (Kruskal-Wallis, χ^2^(2) = 81.01 *p* < 0.01e-16). We then assessed β power across blocks of trials for each task. Tasks were divided in five blocks of trials, with the reward magnitude discrimination task’ blocks containing 14 trials and the probability discounting and probability discrimination task’ blocks containing 26 trials. For the reward magnitude discrimination task, β power was highest in block one and gradually decreased over consecutive blocks (pair-wise Kruskal-Wallis with Bonferroni correction, *p* < 0.01). In both the probability discounting and probability discrimination tasks, β power was also greatest in block 1 and decreased over three successive blocks, with the lowest power observed in blocks 4 and 5 (pair-wise Kruskal-Wallis with Bonferroni correction, *p* < 0.01). Finally for all tasks, β power was low at the very beginning of the session and increased substantially from trial 1 to trial 10 (Pearson’s product moment correlation, reward magnitude discrimination *r* = 0.25, *t*_(498)_ = 5.9, *p* < 0.01e-6; probability discounting *r* = 0.2, *t*_(358)_ = 3.8, *p* < 0.001; probability discrimination *r* = 0.12, *t*_(268)_ = 2.0, *p* = 0.05).

### Contribution of amygdala task-evoked β oscillations to reward expectation in aged rats

To assess whether the task-evoked β oscillations found here play a role beyond indicating a potentially impending reward, we assessed whether trial parameters also contribute to β power. For these analyses, we focused on trials within block 1, which have the highest β power. As shown on Figure 6*C*, we found that β power was slightly, but significantly higher after pressing for the large reward-associated lever, compared to the small reward lever in the reward magnitude task (Kruskal-Wallis χ^2^(1) = 16.6, *p* < 0.001). In contrast, differences in β power following lever press for the probabilistic option in both the probability discounting and probability discrimination tasks did not reach significance (Kruskal-Wallis, probability discounting χ^2^(1) = 2.6, *p* = 0.1; probability discrimination χ^2^(1) = 0.75, *p* = 0.4). Neither the probability of obtaining a reward nor the recent history of wins (Win-Stay/Lose-Shift) had a significant impact on β power (*p* > 0.05). This suggests that that β power may be modulated by the expectation of large reward.

### Correlation between β incidence and movement

Aged rats show slower reactions times in a variety of tasks (Gallagher and Burwell, 1989; Burwell and Gallagher, 1993; Caetano et al., 2012; Roesch et al., 2012a). In this study, however, we did not find an age difference in average reaction times (one-way ANOVA, *F*_(1,11)_ = 0.8, *p* > 0.05). Because the timing of β events can correlate with reaction times in young rats (Leventhal et al., 2012), we addressed the possibility that differences in reaction times may be responsible for the increased β incidence in aged rats, during early session trials. Here, reaction time is defined as the time delay between lever release and nose poking. We specifically evaluated whether early task trials, which are associated with a higher incidence of β epochs and greater β power, had shorter reaction times in aged rats. We measured the average reaction time for each block of trials and found that reaction times were stable, in all tasks performed (reward magnitude discrimination Kruskal-Wallis χ^2^(4) = 3.57, *p* = 0.47, probability discounting Kruskal-Wallis χ^2^(4) = 1.9, *p* = 0.7; probability discrimination Kruskal-Wallis χ^2^(4) = 3.8, *p* = 0.4). The stability of reactions times across trials stands in contrasts with β power which is greater for early session trials and suggests that reaction times do not influence β power in these discrimination learning and decision-making tasks.

Finally, because the power of β oscillations in motor cortex is greatest when subjects hold a position or are immobile, we assessed whether β events also occurred when the rat is immobile. In contrast, we found that task-evoked β events occurred as rats reached the goal location, before reaching immobility. Specifically, the speed of movement during β events was significantly greater than during a baseline period measured immediately before trial onset (speed during task-evoked β 74.2 ± 8.3; speed at baseline 45.62 ± 4.9; Kruskal-Wallis χ^2^(1) = 1026.4, *p* < 0.02e-16). These results suggest that the rats were not immobile during periods of high β power. It is worth noting that these measurements are taken from the LED lights mounted on the brain recording implant, thus primarily reflect head position or orientation.

### β spike coherence, heterogeneous population of BLA neurons participate in transient β oscillations

**Figure 8.**
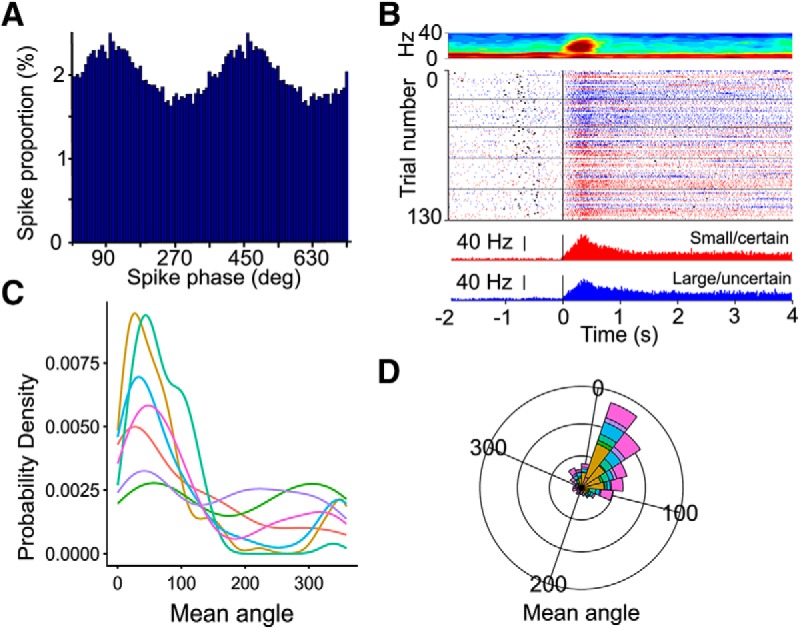
The firing of some BLA cells is phase-locked with β. ***A***, Example firing activity of a BLA neuron phase-locked to β. *y*-axis represents the percentage of spikes at each phase, with the histogram bins for 1 cycle (0–360°) adding to 100%. ***B***, Example session spectrogram in an old rat (top) with raster plot of the firing of an example neuron across trials (center) and corresponding peri-event time histogram of the average spike counts (bottom). The three plots are aligned such that 0 represents the nosepoke (black line). The black ticks in the center raster plot, and to the left of the black nose poke line, represent the lever press timestamps. ***C***, Probability density function of the preferred firing phase on the β cycle, in each aged rat (colors corresponds to each rat in ***D***). ***D***, Polar plot showing the phase relationship between phase-locked BLA neurons and β, in each aged rat (denoted by the different colors).

Neurons of the basal ganglia have previously been shown to be entrained to β oscillations (Leventhal et al., 2012). In the aged rats of the current study, we found that depending on the task, 33% – 45% of neurons became entrained to β oscillations (Rayleigh test, α = 0.05) in late sessions, once β power is established. Specifically, in the reward magnitude discrimination task, 40% of neurons showed phase-locking to β with a mean phase angle of 18.9°; for the probability discrimination task, 33% of neurons were phase-locked to β with a mean phase angle of 17.1°; and for the probability discounting task, 45% of neurons were phase locked to β oscillations and these cells had a mean phase angle of 16.8° (Fig. 8). β Spike coherence could not be assessed in young rats because β events were not evident in this age group.

**Figure 9.**
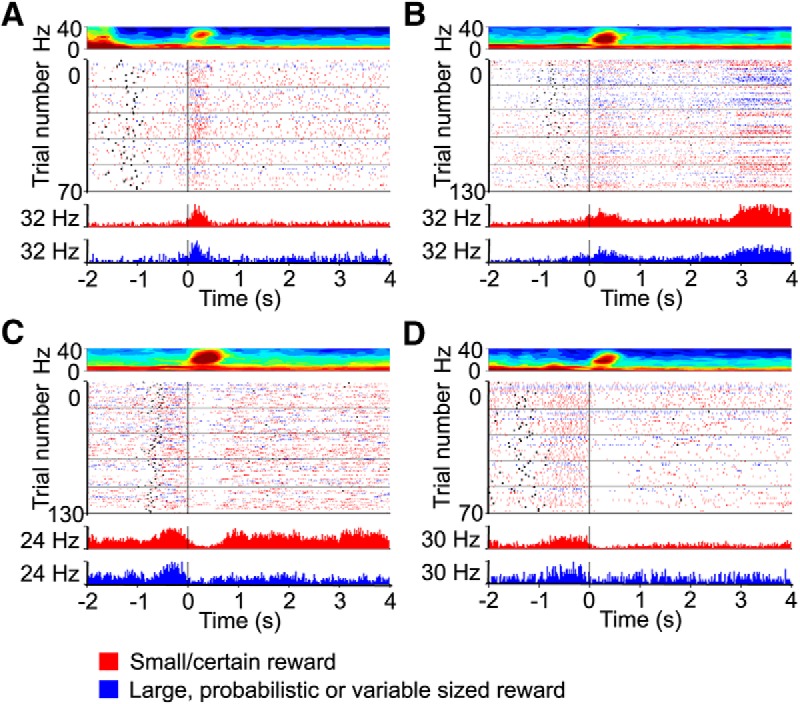
Example firing activity of neurons during task sessions that contained periods of high β power. Four example BLA neurons from aged rats (***A–D***) show the heterogeneity in response pattern, from short bursts of activity, to long sustained activations, as well as drops in firing. The top part of each panel shows the average session spectrogram, the middle portion of each panel shows raster plots of the firing activity during each trial and the bottom portion shows the peri-event time histograms of the average firing activity, averaged by lever choice (red for lever small/certain reward and blue for the variable sized or probabilistic rewards). The three panels are 0 aligned to the first nose poke after lever press. The center raster plot trials are colored as in the bottom panel. The black ticks in the middle raster plot, and to the left of the black nose poke line, represent the lever press timestamps.

Because previous studies indicate that burst firing neurons in the prefrontal cortex may be the ones that are entrained to β phase (Ardid et al., 2015), we investigated whether bursty BLA neurons were also the ones entrained to β in this study. Cell category was inferred by the LV of their spike trains, which measures the degree of burstiness or regularity of the firing pattern. There was a very weak relationship between the burstiness of neurons, and Rayleigh *p* values. This suggests that all BLA neurons contributed similarly to β oscillations, regardless of how bursty their firing pattern was (Pearson correlation *r* = 0.13, *t*_(8691)_ = 11.9, *p* < 2.2e-16, which is a smaller *p* value than Fisher z transform *p* = 0.01 for this sample size). Finally, we assessed the firing pattern of BLA neurons at the first nose poke following lever press (reaching the goal). We found heterogeneous evoked responses profiles following nosepoke into the food area; at the time when β events occur (Fig. 9). Note that these are example taken from 3 neurons that fire coherently with β (Fig. 9*A–C*), and are different from the neurons presented in Figure 8. Figure 9*D* shows the neural activity of a neuron responsive to nosepoke event, but which is not phase-locked to β oscillation, to illustrate the range of response profiles.

We then assessed whether behavior-evoked responses following head entry in the food area were also modulated by β oscillations. We found that the percentage of neurons with responses modulated by the presence of β events within trials was close to chance levels (robust multilinear regression, 6.5% of neurons with *p* < 0.05), suggesting that that the presence of β in a trial did not impact the rate at which BLA neurons fire at head entry. These data suggest that heterogeneous cell populations become entrained with β oscillations in the aged rat’s BLA, and fire in coherence with β oscillations, but not at a different rate during β oscillations. The single-unit activity of BLA neurons during performance of these tasks will be further characterized an upcoming manuscript.

## Discussion

The purpose of this study was to assess whether aging impacts network synchrony or the activity of neurons of the BLA during discrimination learning and decision-making. The primary novel finding is an increase in β power that correlates with behavioral performance in older rats. It is possible that β oscillations in aged rats engage a broad network of brain structures involved in discrimination learning and decision-making, which influences strategy selection during behavioral performance.

### Impact of aging on discrimination learning and decision-making performance in rats

In the present experiment, we found that in the reward magnitude discrimination task, aged rats chose the small reward significantly more often than did young rats. Additionally, aged rats tended to select the small/certain reward option more often in the probability discounting task. Because aging does not impact performance on probability discrimination when the rewards are of equal size in this study or in previous studies (Gilbert et al., 2012; Samson et al., 2015), these results suggest that aging selectively impairs decision-making when different size reward options are involved.

While the behavioral performance results in this study are generally in line with previously published results, they did not completely reproduce the findings presented in a past study (Samson et al., 2015). The present study was designed to examine neural oscillations during discrimination learning and decision-making. Because the protocol was changed compared to the one used in Samson et al. (2015), the data in the two studies are not necessarily at odds with one another. Specifically, the current study used a different number of training sessions and the tasks were interleaved rather than administered sequentially. In fact, consistent with the current study, the protocols used by Gilbert et al. (2012) and Roesch et al. (2012b) also resulted in age differences in reward magnitude discrimination. With respect to the probability discounting task, the present experiment did not extend training past 20 d, and the age effect only arose in the Samson et al. (2015) study when training was carried beyond 30 d. It is likely that a significant age difference would have arisen in the present study if more extensive training had been given.

There are at least two possible interpretations that may account for the age differences in performance on the reward magnitude discrimination task. First, it is possible that lower performance levels in aged rats reflects a reward learning deficit. For example, older adults show faster acquisition speed when using large, as compared to small rewards (Weiler et al., 2008). An alternative hypothesis, however, is that aged rats employ a different strategy than do the younger rats. The strategy that aged rats appear to adopt is one of persistent alternation between levers. One explanation for this would be that the older animals continue to explore possible response options, possibly in anticipation that the contingency will be altered. This possibility could be further explored using different lever-reward associations in rats and in further exploring analogous tasks in humans.

### Impact of aging on activity of single cells and LFPs in the BLA, during discrimination learning and decision-making tasks

Our original hypothesis was that the discrimination learning and decision-making tasks examined in the present study would elicit oscillatory activity within the gamma frequency range (∼40 Hz). Elevated gamma power, for example, has been observed in head-restrained cats during trace-conditioning using visual stimuli (Bauer et al., 2007) and in tone-reward association learning (Popescu et al., 2009), and in rats in probabilistic reward learning (Terada et al., 2013). We did not, however, observe power increases in the gamma frequency range in any of our tasks. Careful examination of the power spectrum from each of these previously published manuscripts, however, does reveal a smaller, but evident increase in power around 15–20 Hz. Each of these manuscripts focus on the larger increases in gamma power or high-frequency oscillations. Thus, these data do not contradict our findings of task- and age-selective increases in β power. One reason that may explain the lack of a change in gamma oscillations in our study is the use of different behavioral procedures (instrumental in our case and Pavlovian in the previous studies). The exact explanation for the lack of gamma power increase in our study, however, remains to be resolved.

The pattern of change in β power found during discrimination learning and decision-making tasks performed in this study bears resemblance to β power changes reported during olfactory learning (for review, see Martin and Ravel, 2014). In both forms of learning tasks, β power builds up early within a daily session to be highest around the 10th to 15th trial (Lowry and Kay, 2007). A second similarity in both types of tasks is that β power develops over days until animals reach criterion (Martin et al., 2004; Cohen et al., 2015). Because of the similarity of β power modulation, it is possible that overlapping network mechanisms can be involved, during discrimination learning and decision-making, as revealed in aged rats.

β Oscillations are prominent in the olfactory bulb and piriform cortex during associative tasks that use olfactory cues (for review, see Martin and Ravel, 2014). These oscillations are well suited to coordinate long-range communication between functional networks and have been implicated in a variety of learning tasks. For example, entorhinal-hippocampal coupling at β frequencies was shown to develop during odor-place association learning (Igarashi et al., 2014). Similarly, ensemble representation in the olfactory bulb, piriform cortex, orbitofrontal cortex and basolateral amygdala have been reported during conditioned odor aversion training (Chapuis et al., 2009). Importantly, the olfactory bulb projects directly to the cortical regions of the amygdala, including the postcortical amygdala and the rostral amygdalopyriform area, which are bidirectionally connected with BLA (Swanson and Petrovich, 1998). Thus, the β oscillations recorded in aged rat’s BLA during performance of the reward discrimination and decision-making tasks in this study may have originated from the olfactory bulb. The recruitment of differential networks could contribute to the behavioral differences found not only in discrimination tasks but also in other tasks involving emotions. In fact, in the present study, the emergence of β oscillations was accompanied by phase-locked single unit responses in over 30% of BLA neurons of old rats. We found that the maximal firing of the neurons followed the peak power of β oscillations, suggesting an interplay between inhibitory and excitatory neurons within this frequency range. Future investigation using multiple recording sites will help identify how and which reward networks are differently recruited in aging.

One important difference between the increased power in the β frequencies in the amygdala versus the olfactory system is the time of occurrence of β oscillations in relation to task events. During olfactory association tasks, β power increases occur during cue-odor sampling, before making a decision (Igarashi et al., 2014). In contrast, the β power increases in our study occurred after the decision was made (lever choice) and well before reward sampling. Because the β oscillations that we found were primarily time locked to head entry into the food cup and well before reward delivery, the data suggest that this is an event of particular relevance to aged rats. Importantly, aged rats show intact thresholds for olfactory cue detection (Yoder et al., 2017). It is possible that aged rats may have formed an association between the residual vanilla scent of the food cup area and upcoming rewards, although not required for accurate performance in any of the tasks administered here.

The age difference in β power in this study is quite striking. Several hypotheses can be offered to explain these results. First, there may be age-related differences in neuron function and the networks that are recruited in these tasks. One candidate that could be partly responsible for an age-related change in oscillatory activity is the known decline of dopaminergic transmission (Bäckman et al., 2010; Li et al., 2010; Eppinger et al., 2011; Darbin, 2012; Li and Rieckmann, 2014; Rieckmann et al., 2016). In fact, reduction in dopamine levels has been shown to increase β power in motor networks (Levy et al., 2002; Weinberger et al., 2006; Mallet et al., 2008; Lemaire et al., 2012; Sharott et al., 2014). Furthermore, L-DOPA therapy in both Parkinson’s patients and dopamine-depleted rats leads to an attenuation of aberrant β -band oscillations (Brown et al., 2001; Lemaire et al., 2012; Gulberti et al., 2015). In addition, older adults also show increased β power (Bruce et al., 2009; Rondina et al., 2015), with inconsistent findings regarding event-related changes in β synchrony between regions (Ho et al., 2012; Vysata et al., 2014). Because short axon cells of the olfactory bulb corelease GABA and dopamine (Liu et al., 2013), it possible that age-related declines in dopamine transmission through this means can also impact β oscillations.

Another possible explanation for our result is that young and aged rats may be using different strategies to solve the discrimination learning and decision-making tasks employed here, leading to the recruitment of different networks.

The increased power of β oscillations only occur in the olfactory system when associations are made between odors and rewards. Furthermore, β oscillations are of low amplitude in anesthetized rats (Lowry and Kay, 2007), and in naïve animals who have not formed associations (Gervais et al., 2007). Blocking cortical afferents to the main olfactory bulb abolishes β oscillations in both the olfactory bulb and the piriform cortex (Martin et al., 2006). Thus, top-down influence from higher order cortical areas is needed for β oscillations to develop and persist, at least during olfactory discrimination learning tasks. One hypothesis that was put forth by Martin and Ravel is that β rhythms are necessary during the acquisition of olfactory discrimination tasks to engage a network of distant brain structures required for specific rule and odor encoding (Martin and Ravel, 2014). Because all young rats in the present study opted for the larger reward option, regardless of probability, they may not have engaged the higher-order brain regions needed for β oscillations to develop. In contrast, aged rats employed what might be considered to be a more task specific strategy. This different strategy may have engaged alternative networks through enhanced β power during performance of these learning tasks. The difference in intrinsic synchrony in BLA may reflect a restructuring of reward circuits during aging.

## Synthesis

Reviewing Editor: Kate Jeffery, University College London Decisions are customarily a result of the Reviewing Editor and the peer reviewers coming together and discussing their recommendations until a consensus is reached. When revisions are invited, a fact-based synthesis statement explaining their decision and outlining what is needed to prepare a revision will be listed below. The following reviewer(s) agreed to reveal their identity: Daoyun Ji.

This study asks how aging impacts amygdalar network activities by investigating age differences in reward processing in rats performing discrimination tasks with varying, and sometimes probabilistic rewards. They looked at how amygdala beta oscillations are involved and found that beta-band (~21 Hz) oscillations emerge with training only in old rats. They also conducted a detailed characterization of beta oscillations in relation to different aspects of task performance in old rats and found evidence that some basal lateral amydalar (BLA) neurons are phase-locked to the beta oscillations.

Concurring with previous reviewers, main concerns include whether the observed betaoriginates within BLA or is volume-transmitted from surrounding olfactory areas, and also what is the behavioral correlate of beta. The authors had tried to address these issues in the revised manuscript, with some success, but the issue remains and it needs to be highlighted in the paper that we cannot rule out that the oscillations come from elsewhere, which does not invalidate the general conclusion about age-related change.

Additionally, the manuscript in its present revised form contains a few important issues, in addition to quite a few typos, that need to be corrected.

- The rationale for looking at aging effects on BLA beta oscillations during reward processing needs to be more clearly stated. Although the authors also looked at theta oscillations, there is arguably as much of a rationale for looking at amygdala gamma in reward processing (e.g. Denis Pare's work). Looking at beta specifically needs to be better justified at the outset (the authors do make this clearer throughout the Results). It's also unclear why functional connectivity between brain areas is stressed in the Abstract and Introduction when activity was only looked at in the amygdala; again, the purpose of the study needs to be made clearer earlier on.

- The authors report age differences in task performance but they don't seem to have replicated their previous results for two of the tasks; this needs addressing. Moreover, the age differences reported in the third task, while significant after re-analysing the data, could be more convincing.

- The authors have characterized beta oscillations extensively during different aspects of the task performance in aged rats. However, they could have made much more of the unit data obtained in the aged rats and as far as I could tell they haven't included any unit analysis from the young rats for comparison, which was the aim of the study. The authors state at the end of the Results that the unit data will be further characterized in a separate paper but it should all be included together with the beta oscillation data here. This would strengthen the paper significantly.

- There are inconsistencies in the description of experimental procedure between the Methods, Fig. 1, and Fig. 1 caption. Was the delay following cue 2 seconds or 3 seconds? Was the lever press period 25 seconds or 20 seconds?

- In Results, Line #370, “a modified version of the decision making task” is inconsistent with Line #209 in the Methods.

- In Lines #637 - 644, this paragraph is very poorly written. In the response letter, the authors said “What was different, was the coherence between cell firing and the phase of beta” and this had been added in this paragraph. However, I do not find any description of data regarding this here at all.
